# The Dynamic Forces of the Homœopathic Remedies

**Published:** 1890-10

**Authors:** 


### THE DYNAMIC FORCES OF THE HOMŒOPATHIC REMEDIES.

(Extract from proceedings of late meeting I. H. A. First day's session.)

Dr. Rushmore—I should like to ask some of our German members whether the word spirit-like or spiritual best gives the sense of the German word used by Hahnemann. Perhaps this would rescue him from the transcendental philosophy applied to him by Dr. Wilson in his paper.

Dr. Wesselhœft—I think it should be spirit-like and not spiritual. This mistake has been made in several translations of the *Organon*, notably in Conrad Wesselhœft's. We should not understand it as pertaining to anything in the line of mysticism. Spirit-like and spiritual are certainly different, yet the difference is very difficult to express. I think that spirit-like refers to the refined and etherial nature of potentized matter, the particles of matter are in a spirit-like form, while the word spiritual excludes altogether the idea of matter.

Dr. Butler—Does not the word spirit-like imply that the whole subject is rather an hypothesis than an actual entity?

Dr. Wesselhœft—There is a point which is interesting to note and which bears upon the action of our remedies, and that is that, although we must select our remedy by the law of similars, the curative action which follows has nothing to do with making another or a similar disease. Dr. Hering used to say that that explanation of Hahnemann's as regards the action of medicine was all wrong; that the indications for a remedy were drawn from the law of similars, but according to that very law the action or effect of the remedy must be contrary. Here we have the line of the action of a disease from one point to another, or the picture of the disease, so to speak, a remedy is selected according to that line, and when these two forces meet they meet as opposites and oppose each other. They do not push along in the same direction, but opposite. Hahnemann's explanation never satisfied me, and I think Dr. Wilson's paper may throw some light upon it.

Dr. Hawley—I should like to ask Dr. Wilson how he escapes the charge of mysticism by simply giving new names to old things. Is not conservation of energy equally mystical to spirit-like force; the difference is only one of terms as it appears to me.

Dr. Butler—Dr. Wilson not being here, of course I do not know how he would answer this question. We must remember that Hahnemann lived sometime ago, and a great many changes have taken place since he died. Indeed, it is astonishing to note how many terms, phrases, and theories there are in medicine to express the same thing. What Hahnemann meant by spirit-like force I do not know, but I suppose that it corresponds very nearly to what is to-day known as energy. Spirit-like force was to him spirit-like in that it had nothing material in it; nowadays we have abolished that word and use the phrase conservation of energy instead.

What he called force we call energy. I think, that perhaps the differences we have in these abstruse subjects are rather in the expression of ideas than in the ideas themselves.

Dr. Hawley—We should be instructed if we had a list of the different words which in times past have been used to express the same idea. We have done away with the word force, and we shall have to do away with the word spirit. I care not whether you call it force, or spirit, or conservation of energy, but I am certain that our art of healing is one of the most substantial things we know anything at all about. We take healing power out of Nux vomica and find it still in the sugar or in the alcohol used to develop it. The curative force of Nux vomica is in the sugar or alcohol; in their potentized state, I have no doubt but that these forces are allied to electricity or magnetism; like forces attracting and dissimilars repelling. The force residing in the potentized medicines perhaps attracts diseased forces similar to itself from the sick, and thus leaves the organism free to go on in its proper way.

Dr. Fincke—The remedy must be similar with the disease in order to cure. This also applies to magnetism, electricity, and all curative forces. Spirit-like does not refer solely to material things, because it refers to that refined dynamic power residing in drugs which when applied according to the law of similars restores the life force of a sick person to its normal action. Still, we must remember that it is the life force which restores health, the remedy only enabling it to do so, probably, by removing some misdirected action. This life force has nothing in common with pure matter, and the force in the remedy which acts upon it has but little in common with pure matter, hence the term spirit-like. It is something like spirit, because it is so refined, rarified and etherialized, yet it is not spirit itself, and I do not think we need find any fault with either the word or the idea.

The life force of Hahnemann has been ruled out by modern philosophers; with them there is no such thing, but the physical form acts by its own properties, matter moves by virtue of something inherent in it, and so they have come to the conclusion that there is no spirit, but Hahnemann's philosophy rests upon a spiritual basis.

Dr. Kent—If we knew what spirit is and vital energy is we would have no difficulty in understanding a good many things that are dark to us now. I have never seen a spirit, and I know it is hard to find words and synonyms with which to express ourselves. We come the nearest to knowing what it is like, by knowing that it is not matter in its usual material, palpable form, and by reflecting upon its extension in our potencies. It is certainly difficult to distinguish between spirit-like and spiritual. Probably the latter word would have been good enough for us were it not supposed, in the popular mind, to have some connection with modern spiritualism, a thing to which we object.

If there were no such thing as modern spiritualism, I do not suppose any one would have raised objections to the word spiritual. Probably as good a thing as we can do on this subject is to understand that we do not understand, and that is arriving at something of a conclusion.

Dr. Carleton—Dilution and attenuation are words I do not like to hear. I have some very deep-rooted prejudices in my make-up, and among the strongest I have are those against these words. Potency and dynamizations are the proper words, and I do not think I heard them in the doctor's paper. I am not much of a philosopher, but allow me to say that I believe in Hahnemann every time.

Dr. Thomson—Some here seem to have trouble as to the existence of spirit, and one gentleman has said he has never seen a spirit. Why, as I look around me in this room, I see a great many spirits. It is the spirit within the man that thinks and acts. It is the spirit that moulds the body and makes the infinite varieties of the human form. Matter, in itself, is dead and inert, and all force comes from the spiritual that vivifies it. The distinction between these two is the distinction between Allopathy and Homœopathy ; the first is dead and lifeless, while the latter is a vivifying force, and its object is to rid this poor body of flesh of its disease by a proper application of that force.

Dr. H. C. Allen—I was much pleased with Dr. Wesselhœft's distinction between spiritual and spirit-like. I think he hit the nail pretty nearly on the head. The terms certain and sure present a similar distinction. I am sure the sun will arise. I am certain it has arisen.

Dr. Butler—In regard to the words dilution and attenuation, neither Dr. Wilson nor I have any apology to make. They are pharmaceutical words and perfectly proper in their place.

At this point Dr. Butler read a paper on the Vital Force. Upon its conclusion, Dr. Fincke said Hahnemann certainly knew much about the subject of this paper, and I wish to refer you to the *Organon*, from the ninth to the fifteenth sections of the fifth edition, where you will find what he made of this subject. He was by no means a theorist, but was eminently of a practical nature; he did not go at all into metaphysical explanations, nor did he attempt to explain what spirit was or what life was. He founded Homœopathy upon the rock and foundation of experience and experiment.

He lays down the life force as the foundation of the healing art, and there is none in the world who can gainsay or oppose that, without stultifying themselves and making a physico-chemical school.

Hahnemann has taught us how, by a simple and beautiful process, we can evolve a spirit-like force from crude substances which is able to influence the vital force and so cure disease. He concluded, from observation, that they must be similar, and thus arose that beautiful generalization, standing upon the firm foundation of experience and experiment, which no one is able to overthrow.

You can't see this spirit-like force, you can't touch it, you can't feel it, but it is there. Has a force ever been seen by any one? You see motion, but you do not see force, you see the effect; and so we cannot see this spirit-like force, but we can see its effect in curing disease.

According to the laws of motion, philosophers say that matter cannot move unless impelled by some force. Therefore, matter is inert and lifeless, and, if inert and lifeless, how can it, in itself, have all these properties and qualities? So there must be a force residing in matter which we develop by the simple and beautiful process of dynamization in our potencies, and which is able to act upon the life force so as to aid it in curing. There is not a crude substance on the earth that has not a medicinal quality, if we can draw it out; even the most inert substance will develop enormous medicinal power if we potentize it.

Dr. Clausen—Where is there a material substance, in nature, whose particles have not been brought together, or are not continually held together by some immaterial spirit-like force? The soul is that immaterial part of man which clothes itself with flesh; it is that spiritual force which draws and holds the atoms together which make the human form. Just so, but without any self-consciousness, there exists, in earths, soils, and plants, an immaterial spirit-like force which holds their particles together and gives each kind of earth, soil, and plant its individual identity. The force, for instance, existing in Silex is always the same, distinct and individual, and by the process of dynamization we bring that force into an accessible condition, and, by the law of similars, apply it to the removal of diseased conditions.

